# Differential potassium channel inhibitory activities of a novel thermostable degradation peptide BmKcug1a-P1 from scorpion medicinal material and its N-terminal truncated/restored peptides

**DOI:** 10.1038/s41598-024-66794-4

**Published:** 2024-07-12

**Authors:** Chenhu Qin, Xuhua Yang, Zheng Zuo, Peixin Yuan, Fang Sun, Xudong Luo, Xiangdong Ye, Zhijian Cao, Zongyun Chen, Yingliang Wu

**Affiliations:** 1 Department of Biochemistry and Molecular Biology, College of Basic Medicine, Hubei University of Medicine, Shiyan, 442000 China; 2 College of Life Sciences, Wuhan University, Wuhan, 430072 China; 3 Center for BioDrug Research, Wuhan University, Wuhan, 430072 China

**Keywords:** Biochemistry, Drug discovery

## Abstract

Thermally stable full-length scorpion toxin peptides and partially degraded peptides with complete disulfide bond pairing are valuable natural peptide resources in traditional Chinese scorpion medicinal material. However, their pharmacological activities are largely unknown. This study discovered BmKcug1a-P1, a novel N-terminal degraded peptide, in this medicinal material. BmKcug1a-P1 inhibited hKv1.2 and hKv1.3 potassium channels with IC_50_ values of 2.12 ± 0.27 μM and 1.54 ± 0.28 μM, respectively. To investigate the influence of N-terminal amino acid loss on the potassium channel inhibiting activities, three analogs (i.e., full-length BmKcug1a, BmKcug1a-P1-D2 and BmKcug1a-P1-D4) of BmKcug1a-P1 were prepared, and their potassium channel inhibiting activities on hKv1.3 channel were verified by whole-cell patch clamp technique. Interestingly, the potassium channel inhibiting activity of full-length BmKcug1a on the hKv1.3 channel was significantly improved compared to its N-terminal degraded form (BmKcug1a-P1), while the activities of two truncated analogs (i.e., BmKcug1a-P1-D2 and BmKcug1a-P1-D4) were similar to that of BmKcug1a-P1. Extensive alanine-scanning experiments identified the bonding interface (including two key functional residues, Asn30 and Arg34) of BmKcug1a-P1. Structural and functional dissection further elucidated whether N-terminal residues of the peptide are located at the bonding interface is important in determining whether the N-terminus significantly influences the potassium channel inhibiting activity of the peptide. Altogether, this research identified a novel N-terminal degraded active peptide, BmKcug1a-P1, from traditional Chinese scorpion medicinal material and elucidated how the N-terminus of peptides influences their potassium channel inhibiting activity, contributing to the functional identification and molecular truncation optimization of full-length and degraded peptides from traditional Chinese scorpion medicinal material *Buthus martensii* Karsch.

## Introduction

Thermally processed *Buthus martensii* Karsch scorpion, a significant traditional Chinese medicinal material, has been utilized in China for over a millennium for treating various neurological disorders^1,2^. However, the effective ingredients for treating diseases in this medicinal material remain unclear. Research on *Buthus martensii* Karsch venom has identified numerous active peptides targeting ion channels, with over 100 characterized to date^3–7^. Notably, traditional scorpion medicinal materials have revealed a rich repository of thermally stable peptides, including full-length potassium toxins and numerous degraded peptides^8^. While the potassium channel inhibiting activities of full-length toxins have been extensively studied^5,9–17^, the potential of degraded peptides from this source remains relatively unexplored, except for peptides such as BmK86-P1, BmKcug2-P1 and BmTX4-P1^12,13,18^.

Potassium channels play critical roles in various human diseases^19–24^. Therefore, further understanding and predicting the potassium channel inhibiting activities of degraded peptides from thermally processed *Buthus martensii* Karsch scorpion are crucial for deciphering the therapeutic components and mechanisms of this medicinal material *Buthus martensii* Karsch.

This study aims to discover new degraded peptides from thermally processed *Buthus martensii* Karsch scorpion and characterize their potassium channel inhibiting activities. Here, we have identified BmKcug1a-P1, a novel N-terminal degraded active peptide, from this medicinal material. We conducted comparisons of the potassium channel inhibiting activities of BmKcug1a-P1 with those of the full-length wild-type toxin BmKcug1 and its two N-terminal truncated peptides (BmKcug1a-P1-D2 and BmKcug1a-P1-D4). Furthermore, we elucidated how the N-terminus of a peptide influences its ability to inhibit potassium channels through extensive alanine-scanning experiments, along with structural and functional analysis. In summary, this study advances the identification of active N-terminal degraded peptides from thermally processed *Buthus martensii* Karsch scorpion and provides new insights for modifying or designing high-affinity peptides by optimizing their N-termini.

## Results

### Discovery and preparation of BmKcug1a-P1 in traditional Chinese scorpion medicinal material

**Figure 1:**
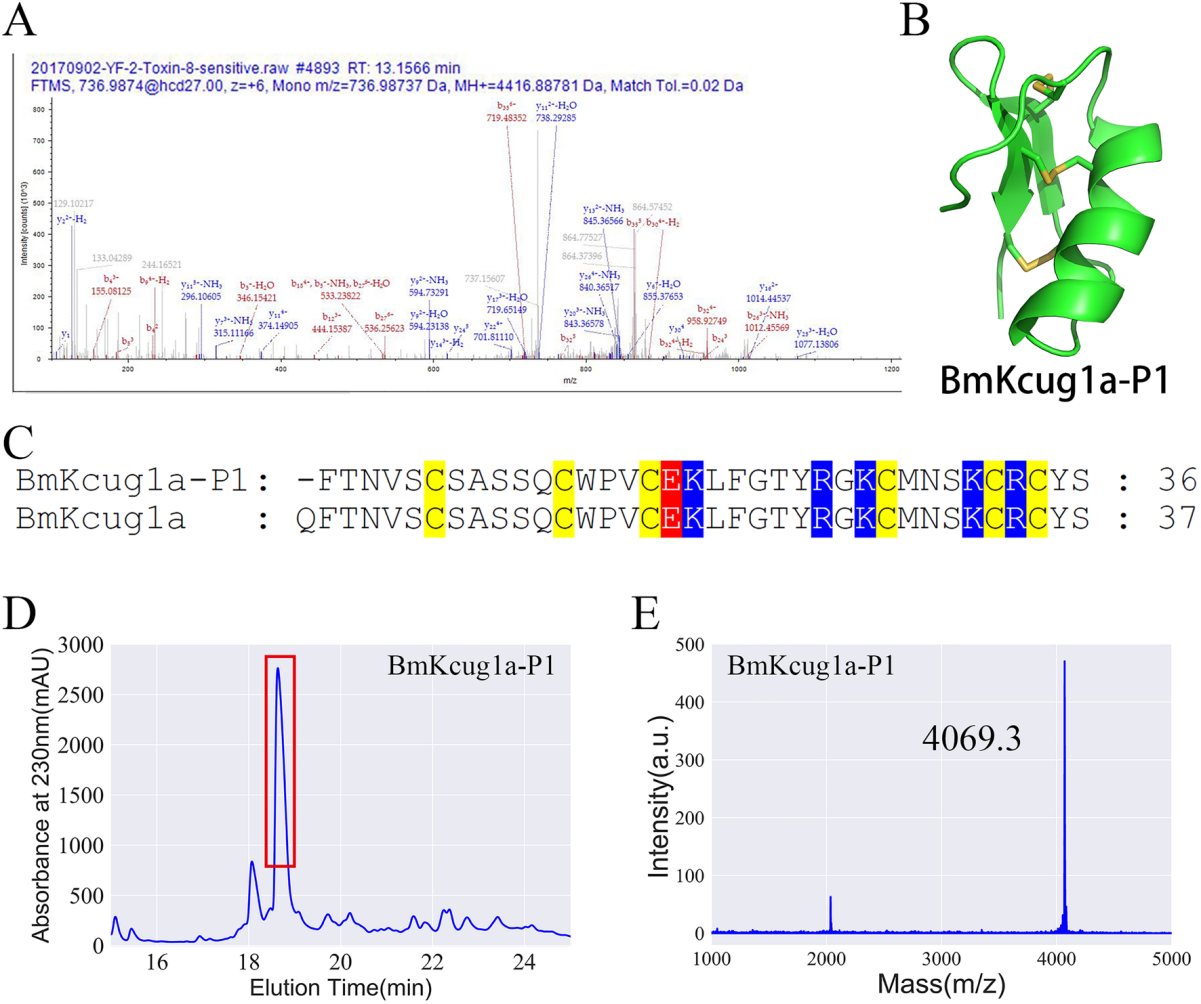
Identification and preparation of the N-terminus degraded peptide BmKcug1a-P1 from thermally processed *Buthus martensii* Karsch scorpion. (**A**) BmKcug1a-P1 was identified in thermally processed *Buthus martensii* Karsch scorpion by MS/MS spectra analysis. The series of b-ion and y-ion are shown in red and blue, respectively. (**B**) The 3D structure of BmKcug1a-P1 was modeled using the SWISS-MODEL server based on the BmKcug1a template (PDB code: H2ER22). (**C**) Sequence alignment of BmKcug1a-P1 and wild-type toxin BmKcug1a. The yellow, blue and red are the background colors of cysteines, basic residues and acidic residues, respectively. (**D**) Recombinant BmKcug1a-P1 was purified using high-performance liquid chromatograph. The fraction indicated by the red box contained BmKcug1a-P1. (**E**) The molecular weight of purified BmKcug1a-P1 was identified by MALDI-TOF–MS, which corresponded to its calculated value.

It has been reported that there are a large number of degraded peptides in thermally processed *Buthus martensii* Karsch scorpion. However, we still know very little about these degraded peptides. Here, we identified a novel degraded peptide, BmKcug1a-P1, in thermally processed *Buthus martensii* Karsch scorpion through mass spectrometric analyses. Compared with the full-length wild-type toxin BmKcug1a, BmKcug1a-P1 is missing one amino acid at the N-terminal region (Fig. 1C). The b-ions and y-ions detected in the spectra confirmed the presence of the peptide BmKcug1a-P1 (Fig. 1A). The 3D structure of BmKcug1a-P1 was predicted using SWISS-MODEL, revealing that BmKcug1a-P1 could form a stable motif with a cysteine-stabilized α-helical and β-sheet (CSαβ) fold (Fig. 1B).

Based on the amino acid sequence of BmKcug1a-P1, we constructed a prokaryotic expression vector for BmKcug1a-P1. The vectors of BmKcug1a-P1 were transformed into *E. coli* Rosetta (DE3) cells and the peptides were induced for overexpression using IPTG. The fusion proteins were purified by Ni^2+^ affinity chromatography, then the peptides were cleaved from His tag using recombinant enterokinase. High-performance liquid chromatography (HPLC) was utilized to further purify and isolate the digested peptide on a C18 column (10 × 250 mm, 5 µm) using a linear gradient of 5–95% acetonitrile with 0.1% trifluoroacetic acid (TFA) over 60 min at a constant flow rate of 4 mL/min. Manual collection of fractions was performed at 18–19 min (Fig. 1D). Approximately 0.5–1 mg of purified peptide was obtained from 1 L of bacterial culture. The molecular weight of BmKcug1a-P1 was analyzed by MALDI-TOF–MS, which corresponded to its calculated value of 4069.7 Da (Fig. 1E). This result demonstrates the successful preparation of recombinant BmKcug1a-P1.

### Pharmacological properties of BmKcug1a-P1

**Figure 2:**
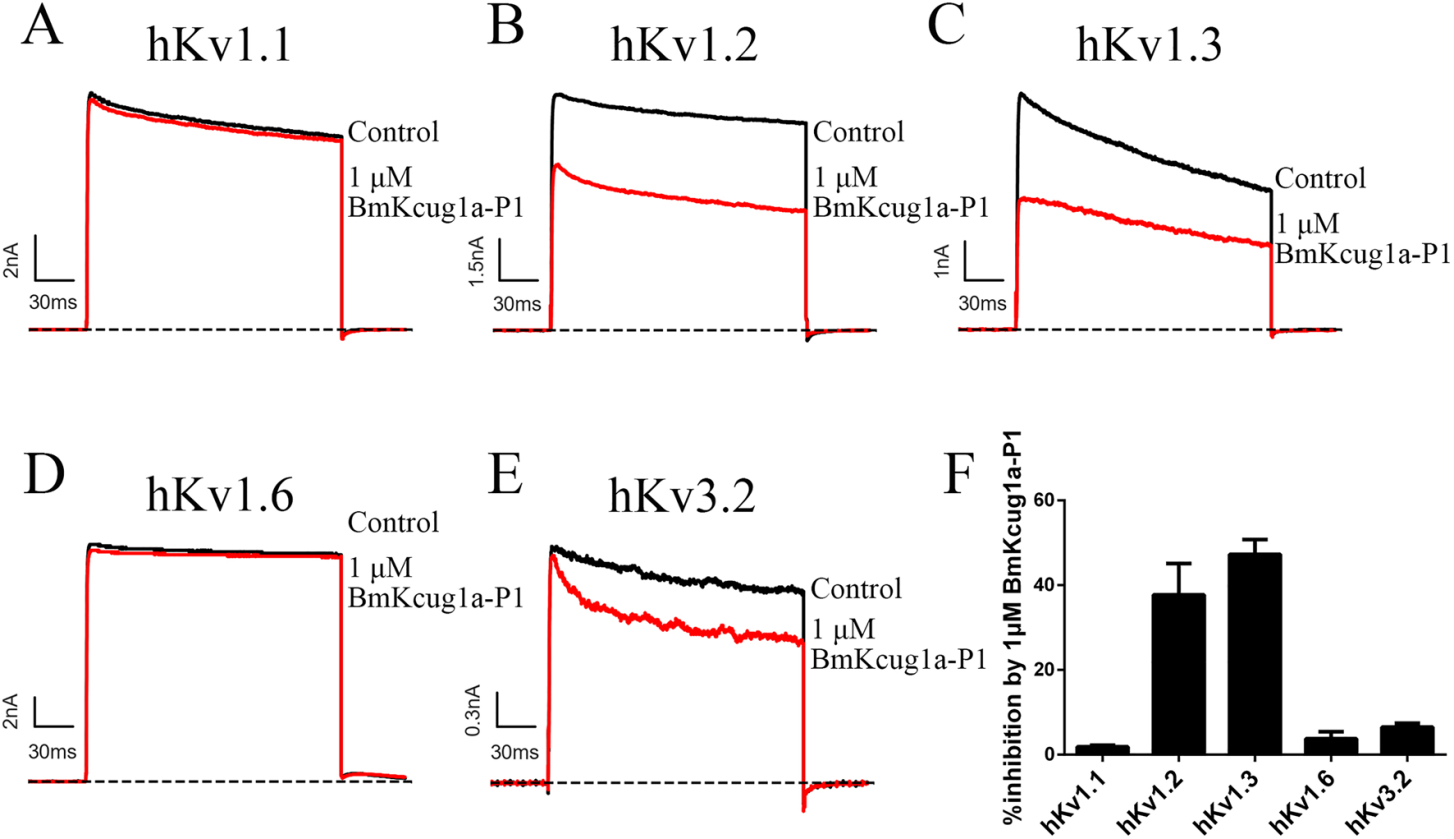
Inhibitory Effects of BmKcug1a-P1 on Potassium Channels. (**A**–**E**) Blocking effects of 1 μM BmKcug1a-P1 on potassium currents mediated by hKv1.1, hKv1.2, hKv1.3, hKv1.6 and hKv3.2 channels. (**F**) The inhibitory rates of BmKcug1a-P1 on hKv1.1, hKv1.2, hKv1.3, hKv1.6 and hKv3.2 channels. Each channel was tested at least three times (n ≥ 3). The results are shown as the mean ± SE.

**Figure 3:**
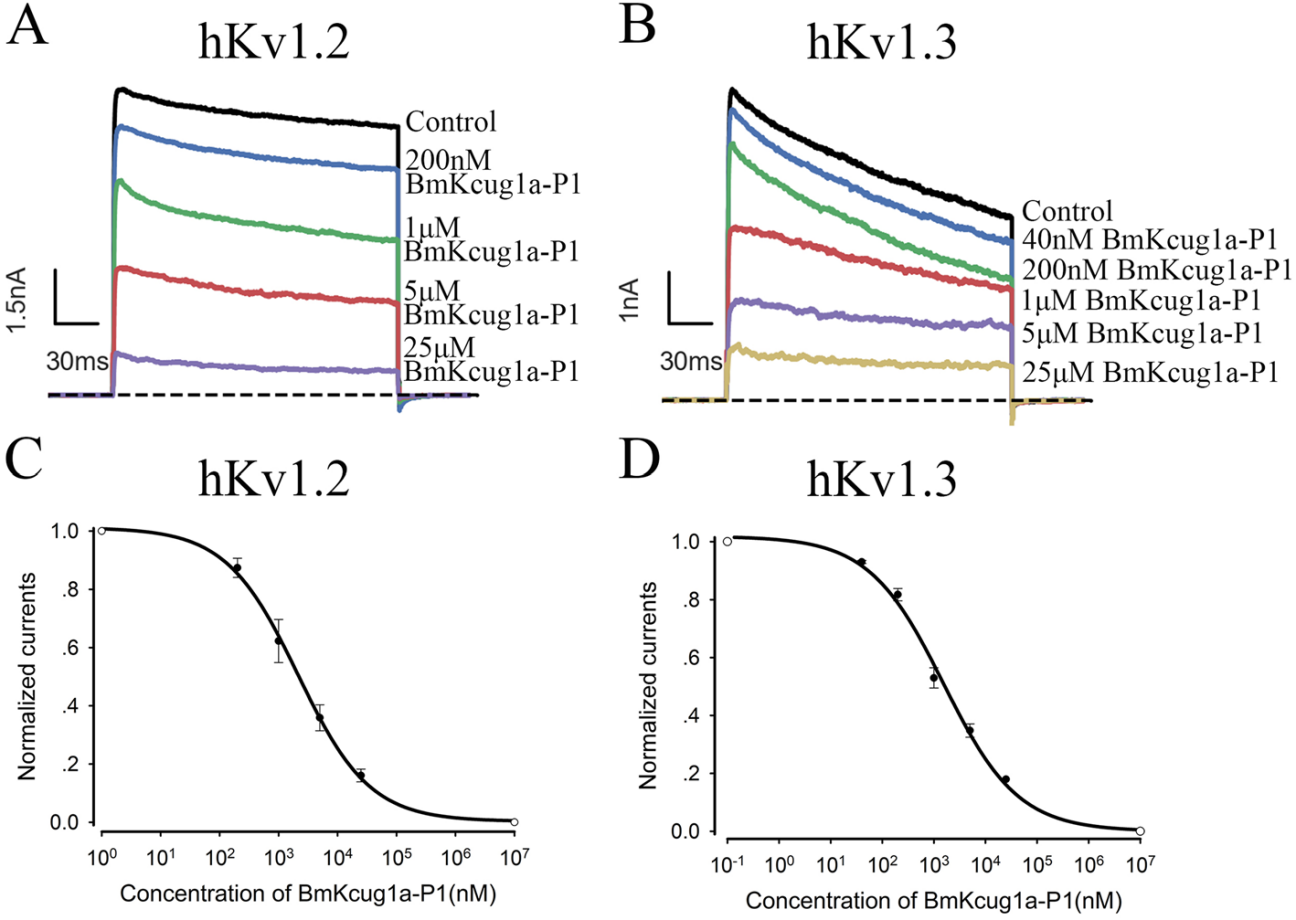
BmKcug1a-P1 inhibited the potassium currents mediated by hKv1.2 and hKv1.3 channels in concentration-dependent manner. (**A**) At concentrations of 200 nM, 1 μM, 5 μM and 25 μM, BmKcug1a-P1 inhibited 12.6 ± 3.3%, 37.7 ± 7.4%, 64.1 ± 4.5% and 83.9 ± 2.2% of hKv1.2 channel current, respectively. (**B**) At concentrations of 40 nM, 200 nM, 1 μM, 5 μM and 25 μM, BmKcug1a-P1 inhibited 7.0 ± 0.5%, 18.3 ± 2.1%, 47.0 ± 3.5%, 65.2 ± 2.3% and 82.0 ± 0.6% of hKv1.3 channel current respectively. (**C**) The average normalized potassium currents of hKv1.2 channel were suppressed by BmKcug1a-P1 at different concentrations, indicating that the IC_50_ value is 2.12 ± 0.27 μM. (**D**) The average normalized potassium currents of hKv1.3 channel were suppressed by BmKcug1a-P1 at different concentrations, indicating that the IC_50_ value is 1.54 ± 0.28 μM.

To further investigate whether BmKcug1a-P1 is an active peptide in thermally processed *Buthus martensii* Karsch scorpion capable of inhibiting currents of potassium channels, we tested it on five important potassium channels subtypes (i.e., hKv1.1, hKv1.2, hKv1.3, hKv1.6 and hKv3.2). The whole-cell patch clamp technique was applied to measure the inhibitory effect of 1 μM BmKcug1a-P1 on potassium channel currents. The results showed that 1 μM BmKcug1a-P1 could inhibit 1.9 ± 0.3%, 37.7 ± 7.4%, 47.3 ± 3.5%, 3.8 ± 1.6%, 6.5 ± 0.9% of potassium currents mediated by hKv1.1, hKv1.2, hKv1.3, hKv1.6 and hKv3.2, respectively (Fig. 2), which indicated that BmKcug1a-P1 is a novel inhibitor of hKv1.2 and hKv1.3 channels. Based on this, we further determined the IC_50_ values of BmKcug1a-P1 on hKv1.2 and hKv1.3 channels from the dose-dependent relationship. The results indicated that BmKcug1a-P1 can inhibit hKv1.2 and hKv1.3 channels with IC_50_ values of 2.12 ± 0.27 μM and 1.54 ± 0.28 μM, respectively (Fig. 3).

### Effects of N-terminus alterations on BmKcug1a-P1’s potassium channel inhibiting activities

**Figure 4:**
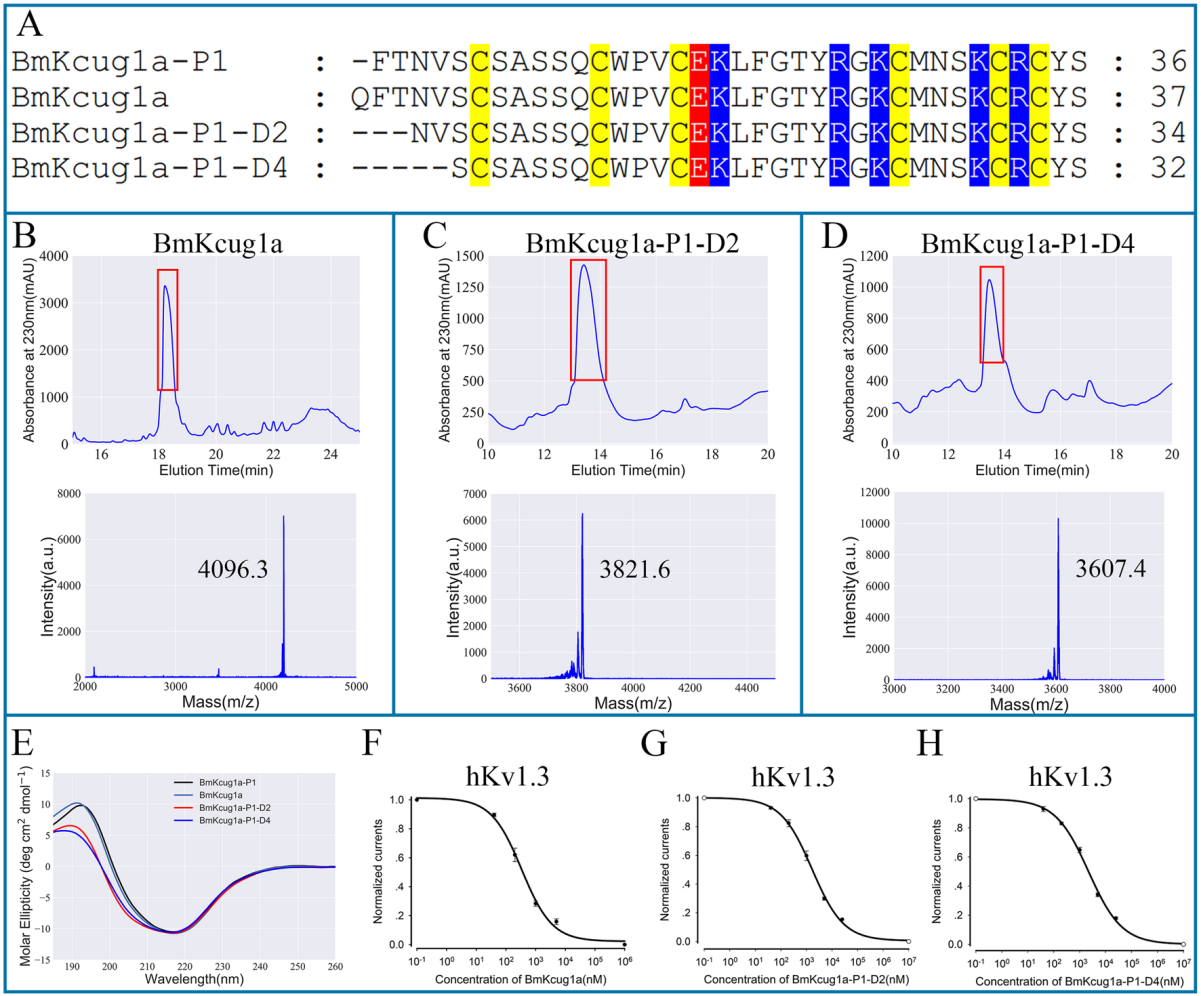
The potassium channel inhibiting activities of BmKcug1a-P1 and its three analogs. (**A**) Sequence alignment of BmKcug1a and its three analogs. The yellow, blue and red are the background colors of cysteines, basic residues and acidic residues, respectively. (**B**–**D**) Preparation of three analogs of BmKcug1a-P1. The peptides were purified by high-performance liquid chromatograph and then identified by MALDI-TOF-MS. (**E**) Circular dichroism spectra of BmKcug1a-P1 and its three analogs. (**F**) The average normalized potassium currents of hKv1.3 channel were suppressed by BmKcug1a at different concentrations, indicating that the IC_50_ value is 0.34 ± 0.08 μM. (**G**) The average normalized potassium currents of hKv1.3 channel were suppressed by BmKcug1a-P1-D2 at different concentrations, indicating that the IC_50_ value is 1.66 ± 0.18 μM. (**H**) The average normalized potassium currents of hKv1.3 channel were suppressed by BmKcug1a-P1-D4 at different concentrations, indicating that the IC_50_ value is 2.21 ± 0.28 μM.

Compared with the full-length wild-type toxin BmKcug1a, BmKcug1a-P1 lacks one amino acid at the N-terminal region. Pharmacological experiments showed that BmKcug1a-P1 remains an active peptide acting on potassium channels. To clarify the influence of the peptide N-terminus on its potassium channel inhibiting activities, three analogs of BmKcug1a-P1 including the wild-type toxin BmKcug1a and two truncated analogs (i.e., BmKcug1a-P1-D2 and BmKcug1a-P1-D4) were prepared. The two truncated analogs retained the disulfide bond-stabilized α-helical and β-sheet motifs while missing two and four amino acids at the N-terminus compared with BmKcug1a-P1, respectively (Fig. 4A). Firstly, BmKcug1a, BmKcug1a-P1-D2 and BmKcug1a-P1-D4 were successfully obtained by prokaryotic expression (Fig. 4B–D). They were purified by the high-performance liquid chromatography and identified by mass spectrometry (Fig. 4B–D). The overall structural topologies of BmKcug1a, BmKcug1a-P1-D2 and BmKcug1a-P1-D4 were further analyzed by CD spectroscopy. The spectra were recorded at a range of wavelengths from 180 to 260 nm in water at RT (~ 25 °C). The result indicates that the structures of BmKcug1a, BmKcug1a-P1-D2 and BmKcug1a-P1-D4 had no significant changes compared to BmKcug1a-P1 (Fig. 4E). Subsequently, BmKcug1a, BmKcug1a-P1-D2 and BmKcug1a-P1-D4 were applied to potassium channel hKv1.3 through the patch clamp technique to further investigate their pharmacological properties. The results showed that BmKcug1a, BmKcug1a-P1-D2 and BmKcug1a-P1-D4 could decrease the currents of hKv1.3 channel in a dose-dependent manner. IC_50_ values for BmKcug1a, BmKcug1a-P1-D2 and BmKcug1a-P1-D4 were determined by fitting dose–response curves with a Hill equation to assess their inhibitory effects on the hKv1.3 channel. Interestingly, compared with BmKcug1a-P1, BmKcug1a exhibited much better channel inhibiting activity towards hKv1.3 channel with an IC_50_ of 0.34 ± 0.08 μM, while BmKcug1a-P1-D2 and BmKcug1a-P1-D4 exhibited similar activities towards the hKv1.3 channel with IC_50_ values of 1.66 ± 0.18 μM and 2.21 ± 0.28 μM, respectively (Fig. 4F–H). This result revealed that the N-terminus of peptides has different effects on their potassium channel inhibiting activity.

### Binding interface and functional sites of BmKcug1a-P1 revealed by alanine-scanning mutagenesis

**Figure 5:**
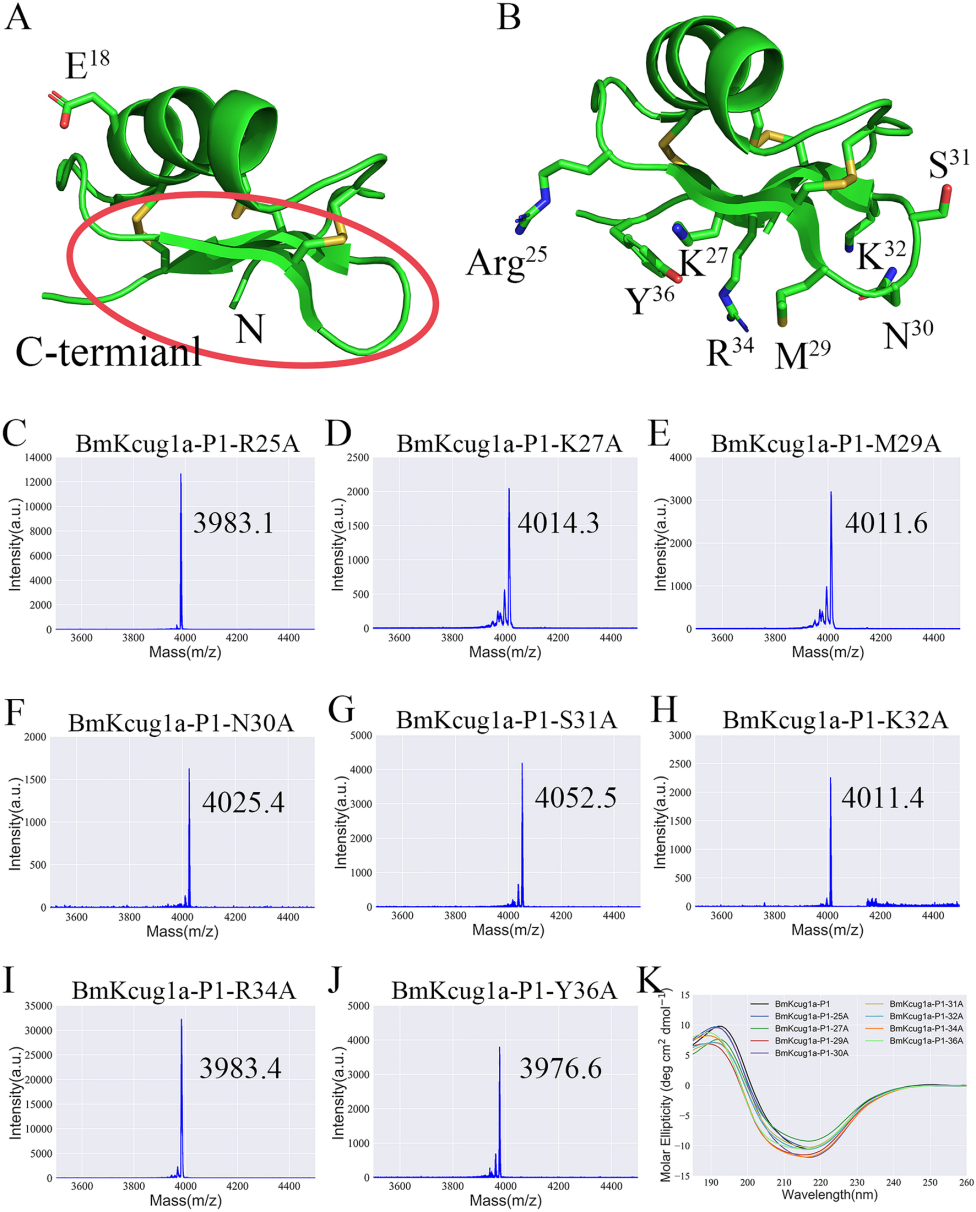
Preparation and overall structural topologies analysis of eight mutant peptides of BmKcug1a-P1. (**A**) The 3D structure of BmKcug1a-P1, in which the acidic amino acid was highlighted and the predicted bonding interface to interact with hKv1.3 channel was enclosed by the red box. (**B**) The 3D structure of BmKcug1a-P1, in which the eight amino acid residues selected to mutate to alanine were highlighted. (**C**–**J**) The purified mutant peptides identified by MALDI-TOF–MS. The molecular weights of eight mutant peptides were consistent with their calculated values. (**K**) The overall structural topologies of eight mutant peptides of BmKcug1a-P1 were analyzed by Circular dichroism spectra.

**Figure 6:**
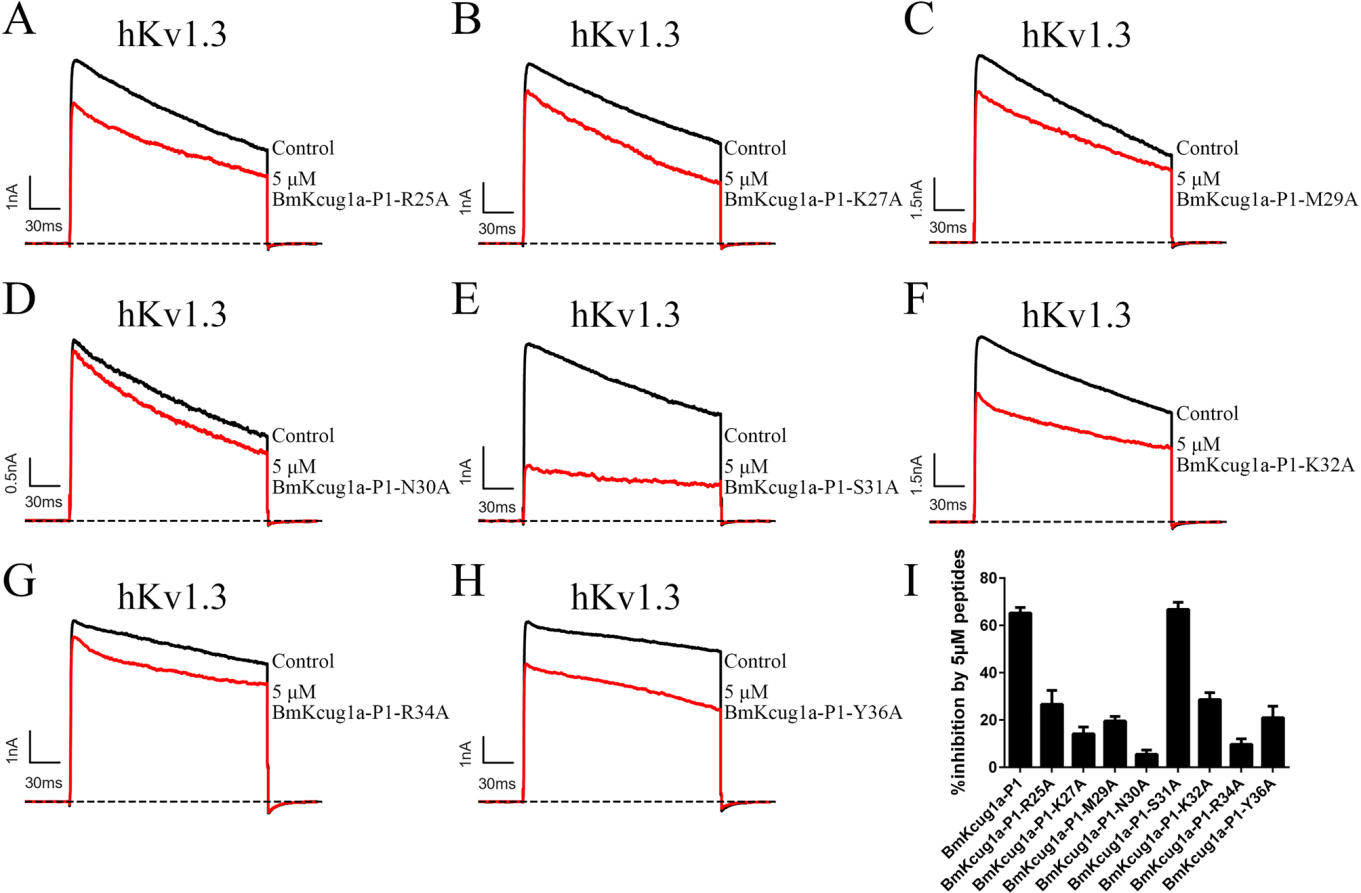
Potassium channel inhibiting activities of the eight mutant peptides of BmKcug1a-P1 on hKv1.3 channel. (**A**–**H**) Traces of potassium currents are shown in the absence and presence of the mutant peptides at the concentration of 5 μM. The hKv1.3 channel was inhibited by BmKcug1a-R25A (**A**), BmKcug1a-K27A (**B**), BmKcug1a-M29A (**C**), BmKcug1a-N30A (**D**), BmKcug1a-S31A (**E**), BmKcug1a-K32A (**F**), BmKcug1a-R34A (**G**) and BmKcug1a-Y36A (**H**). (**I**) The inhibitory rates of 5 μM mutant peptides on currents mediated by hKv1.3 channel.

To elucidate why the N-terminus of peptides has different effects on its potassium channel inhibiting activity, we further investigated the bonding interface and functional sites of BmKcug1a-P1 involved in the interaction with the hKv1.3 channel. According to that the distribution of acidic amino acid residues in toxins would induce the orientation of binding interface^15–17^, the region enclosed by the red box was predicted to be the bonding interface of BmKcug1a-P1 to interact with hKv1.3 channel (Fig. 5A). To validate this prediction and identify the functional sites of BmKcug1a-P1, eight mutant peptides of BmKcug1a-P1 were designed, in which eight residues (i.e., Arg25, Lys27, Met29, Asn30, Ser31, Lys32, Arg34 and Tyr36) located in bonding interface were individually mutated to alanine residues (Fig. 5B). These eight mutant peptides were prepared by prokaryotic expression and further analyzed by mass spectrometry (Fig. 6C–J). The measured molecular weights of mutant peptides were consistent with their calculated values (Fig. 5C–J). There are no significant changes of CD spectra between the eight mutants (i.e., BmKcug1a-P1-R25A, BmKcug1a-P1-K27A, BmKcug1a-P1-M29A, BmKcug1a-P1-N30A, BmKcug1a-P1-S31A, BmKcug1a-P1-K32A, BmKcug1a-P1-R34A, and BmKcug1a-P1-Y36A) and the BmKcug1a-P1 peptide, indicating that the overall structural topologies of the eight mutants were the same to that of BmKcug1a-P1 (Fig. 5K).

Subsequently, the blocking activities of these eight mutant peptides on the hKv1.3 channel were evaluated using patch clamp technology. BmKcug1a-P1-N30A and BmKcug1a-P1-R34A exhibited a dramatic drop in affinity for the hKv1.3 channel. At 5 μM concentration, BmKcug1a-P1-N30A and BmKcug1a-P1-R34A inhibited only 5.5 ± 1.8% and 9.7 ± 2.3% of the potassium currents mediated by the hKv1.3 channel, respectively (Fig. 6D,G,I ). This result indicated that Asn30 and Arg34 of BmKcug1a-P1 were the key functional residues to interact with the hKv1.3 channel. Additionally, the activity of mutant peptides (i.e., BmKcug1a-P1-R25A, BmKcug1a-P1-K27A, BmKcug1a-P1-M29A, BmKcug1a-P1-K32A and BmKcug1a-P1-Y36A) on the kv1.3 channel also decreased, indicating that these residues (i.e., Arg25, Lys27, Met29, Lys32 and Tyr36) were indeed located at bonding interface to interact with the hKv1.3 channel (Fig. 6A,B,C,F,H,I).

### The position of the N-terminus dictates the influence of N-terminus alterations on BmKcug1a-P1 activity

**Figure 7:**
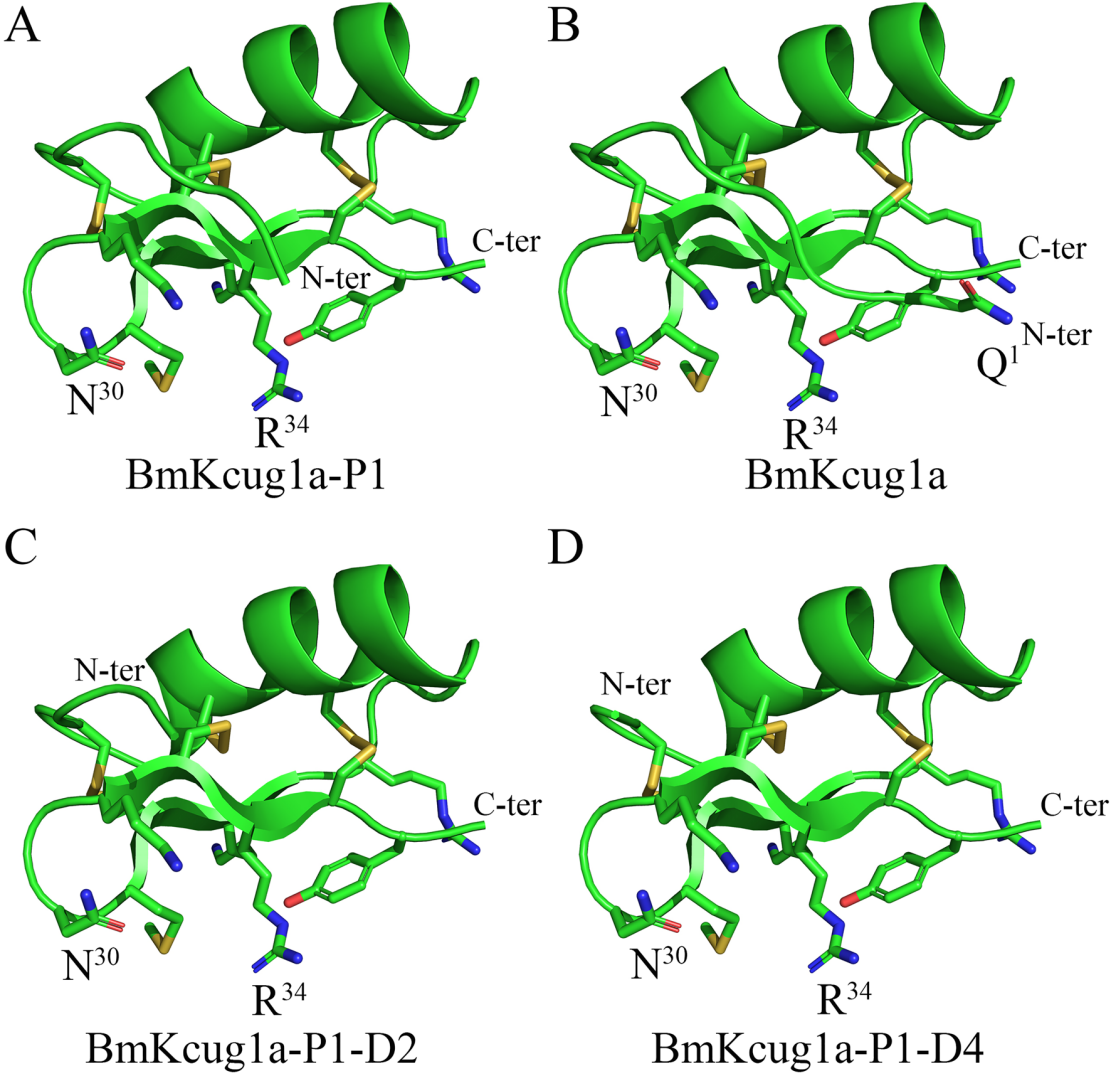
The structure analysis of BmKcug1a-P1 and its three analogs. (**A**) The structure of BmKcug1a-P1, highlighting the N-terminus and the binding interface. (**B**) The structure of BmKcug1a, highlighting the N-terminus and the binding interface. (**C**) The structure of BmKcug1a-P1-D2, highlighting the N-terminus and the binding interface. (**D**) The structure of BmKcug1a-P1-D4, highlighting the N-terminus and the binding interface.

The differing potassium channel inhibitory activities of four peptides (BmKcug1a-P1, BmKcug1a, BmKcug1a-P1-D2, and BmKcug1a-P1-D4) indicate that the N-terminus of each peptide has varying effects on its potency. We conducted further analysis on the positioning of their N-terminal structures following identification of the bonding interface and functional sites of BmKcug1a-P1 (Fig. 7). Our findings revealed that the N-terminus of BmKcug1a is positioned at the bonding interface for interaction with the potassium channel, whereas the N-terminus of BmKcug1a-P1 is not involved in this interaction. This distinction explains why BmKcug1a exhibits significantly stronger potassium channel inhibitory activity on the hKv1.3 channel compared to BmKcug1a-P1, while the two truncated analogs of BmKcug1a-P1 (BmKcug1a-P1-D2 and BmKcug1a-P1-D4) demonstrate similar potassium channel inhibitory activities on the hKv1.3 channel as BmKcug1a-P1.

## Discussion

Thermally processed *Buthus martensii* Karsch scorpion is a significant traditional Chinese medicinal material, widely employed in the treatment of various diseases including epilepsy and rheumatoid arthritis^1^. In recent decades, this medicinal material has garnered increasing attention, leading to numerous studies investigating its pharmacological effects on different diseases^12–14^. However, research on the active constituents of this medicinal material for disease treatment has not made substantial progress.

As it is well-known, this medicinal material is derived from *Buthus martensii* Karsch, and the composition of the venom from *Buthus martensii* Karsch has been elucidated to contain a significant number of channel-inhibiting neurotoxin peptides^3–5^. Increasing evidence suggests that ion channels are closely associated with various diseases such as epilepsy, pain, and dementia^7,16,17,25^. Therefore, our attention was drawn several years ago to investigate whether there are bioactive peptides in thermally processed *Buthus martensii* Karsch scorpion derived from these venom-gland neurotoxin peptides, leading us to discover 22 full-length potassium channel-inhibiting neurotoxin peptides along with a substantial amount of degraded peptides in this medicinal material^8,12,18^. Extensive research has been conducted on the potassium channel inhibiting activities of most full-length potassium toxin peptides during the past five decades studying scorpion venom from *Buthus martensii* Karsch^5,26–33^. However, there remains a significant knowledge gap regarding the potassium channel inhibiting activities of these degraded peptides and how peptide degradation influences their functions, which significantly hampers the identification of the effective medicinal components in thermally processed *Buthus martensii* Karsch scorpion.

**Figure 8:**
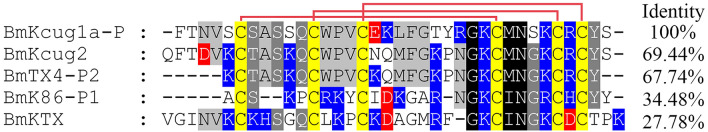
Sequence alignment between BmKcug1a-P1 and the bioactive peptides identified in traditional Chinese scorpion medicinal material.

In this study, a novel degraded peptide, BmKcug1a-P1, was identified from thermally processed *Buthus martensii* Karsch scorpion. Pharmacological experiments revealed that despite missing one residue at its N-terminus compared to wild-type toxin BmKcug1a, BmKcug1a-P1 still exhibited inhibitory effects on potassium channels hKv1.2 and hKv1.3 with IC_50_ values of 2.12 ± 0.27 μM and 1.54 ± 0.28 μM, respectively. Four thermostable potassium channel-inhibiting peptides have been identified in thermally processed *Buthus martensii* Karsch scorpion, such as BmKcug2, BmK86-P1, BmTX4-P1, and BmKTX. Notably, BmKcug1a-P1 shows low sequence similarity to these peptides identified from this medicinal material, indicating molecular diversity of bioactive peptides derived from thermally processed *Buthus martensii* Karsch scorpion (Fig. 8). This highlights the importance of exploring new degraded peptides in thermally processed *Buthus martensii* Karsch scorpion and their potential pharmacological activities, as they may contribute to uncovering new bioactive peptides and new therapeutic effects associated with this material.

In addition, this result has piqued our interest in investigating the impact of peptide N-termini on potassium channel inhibitory activities of peptides. Thus, we prepared wild-type toxin BmKcug1a and two truncated peptides designed from BmKcug1a-P1 and then these three peptides were applied to whole-cell patch clamp experiments to assess their pharmacological activity. Intriguingly, the inhibitory activity of BmKcug1a on hKv1.3 channel was significantly enhanced compared to that of BmKcug1a-P1, whereas the activities of the two truncated analogs on hKv1.3 channels resembled that of BmKcug1a-P1. This outcome suggests that the N-terminus of peptides exerts varying degrees of influence on their channel inhibiting activity. This underscores the complexity of peptide structure–activity relationships and highlights the importance of understanding how N-terminal degradation impact peptide function.

Through further identification of the bonding interface and functional sites of BmKcug1a-P1, the molecular mechanism underlying the impact of the peptide's N-terminus on channel inhibiting activity was elucidated. Experimental findings revealed that Asn30 and Arg34 of BmKcug1a-P1 are pivotal residues for interaction with the hKv1.3 channel. Based on this insight and structural analysis of BmKcug1a-P1 and its three analogs (Fig. 7), it becomes clear how the N-terminus of peptides influences their channel inhibiting activities. The Gln^1^ in N-terminus of BmKcug1a is located at the bonding interface which is crucial for interacting with potassium channels. Consequently, the absence of Gln^1^ significantly reduces affinity of BmKcug1a-P1 for the hKv1.3 channel. Conversely, since the N-terminal residues (i.e., Phe^2^, Thr^3^, Asn^4^ and Val^5^) of BmKcug1a-P1 don’t participate in the bonding interface for interacting with potassium channels, the two truncated analogs of BmKcug1a-P1 exhibit similar pharmacological activities on hKv1.3 channel compared to BmKcug1a-P1. These results underscore that the impact of the N-terminus of peptides on their potassium channel inhibiting activities primarily depends on whether the N-terminus resides located in the bonding interface. This study enhances our understanding of N-terminal degradation peptides from this medicinal material and sheds light on whether the N-termini of peptides govern their pharmacological activities.

The proteomics analysis conducted previously by us on thermally processed *Buthus martensii* Karsch scorpion revealed the presence of numerous peptides with various degrees of N-terminal degradation. However, it remained unclear whether these degraded peptides retained their potassium channel inhibiting activities. The present study introduces a potential strategy for analyzing the function of the N-terminal degraded peptides and will advance pharmacological activity research on the natural N-terminal degraded peptides. Moreover, the study presents new insights into designing shortened mini peptides by adjusting the N-terminus regions of natural full-length peptides and partially degraded peptides. These mini peptides may offer similar pharmacological effects with advantages such as lower molecular weight, reduced cost, and enhanced drug accessibility. Altogether, this study expands our understanding of N-terminal degradation peptides derived from thermally processed *Buthus martensii* Karsch scorpion and provides new insights into modifying or designing high-affinity peptides by manipulating their N-terminus.

## Methods

### Identification of BmKcug1a-P1 from the thermally processed *Buthus martensii* Karsch scorpion

The traditional Chinese scorpion medicinal materials were procured from Tong Ren Tang, a renowned pharmacy known for supplying a variety of materials used in traditional Chinese medicine. The N-terminal degraded peptide BmKcug1a-P1 was discovered in this medicinal material by HCD MS/MS technology according to our previously reported procedure^8^.

### Construction of recombinant expression vectors of BmKcug1a-P1, its analogs and its mutant peptides

The DNA fragments encoding BmKcug1a-P1 were obtained using the classical overlapping PCR strategy using the designed primers^10^. Then the DNA fragments were inserted into the expression vector pET-32a. Recombinant expression vectors of the analogs and mutant peptides were generated via directed mutagenesis using specific primers based on BmKcug1a-P1 expression vectors. Verification of the recombinant plasmids was carried out through sequencing.

### Expression, purification and identification of peptides

Initially, recombinant expression vectors of peptides were transformed into E. coli Rosetta (DE3) cells. The transformed bacteria were cultured in LB medium supplemented with 50 μg/ml ampicillin at 37 °C. Upon reaching the logarithmic growth phase, protein expression was induced by adding 1 mM isopropyl β-D-thiogalactopyranoside (IPTG) and continued at 25 °C for 12–15 h. Subsequently, bacterial cells were harvested, resuspended in chilled 20 mM imidazole buffer (pH 7.9), and lysed using ultrasonic bath. Fusion proteins were purified using Ni^2+^ affinity chromatography, followed by cleavage of peptides from the His tag using recombinant enterokinase (Sangon Biotech, Shanghai, China) at 25 °C for 12 h. Finally, high-performance liquid chromatography (HPLC) was employed for further purification and isolation of the digested proteins on a C18 column (10 × 250 mm, 5 µm), using a linear gradient of 5–95% acetonitrile with 0.1% trifluoroacetic acid (TFA) over 60 min at a constant flow rate of 4 mL/min. Identification of purified peptides was performed using MALDI-TOF–MS.

### Source of potassium channel expression vectors

The cDNA fragments encoding potassium channels were provided by others as previously described^12^. The cDNA encoding hKv1.1 channel and hKv1.3 channel were cloned into the pCDNA3.1(+) vector. The cDNA encoding hKv1.2 channel, hKv1.6 channel and hKv3.2 channel were inserted into the pIRES2-EGFP vector.

### Cell culture and overexpression of potassium channels

HEK293T cell lines were obtained from the BeNa Culture Collection (Xinyang, China) and maintained in DMEM (Dulbecco’s Modified Eagle Medium, Gibco, Pittsburgh, PA, USA) supplemented with 10% fetal bovine serum (FBS, Gibco) and 1% penicillin/streptomycin (Gibco). Cells were passaged using Pancreatin (Gibco). For transfection experiments, HEK293T cells were seeded in appropriate culture vessels and allowed to reach 70–80% confluency. The expression vectors of pEGFP-N1 and potassium channels were co-transfected into HEK293T cells using ExFect Transfection Reagent (Vazyme, Nanjing, China) following its manufacturer’s instructions. Briefly, plasmid DNA and transfection reagent were mixed in serum-free medium, incubated for 15–30 min at room temperature to allow complex formation, and then added to the cells. After 24–48 h transfection, the single positive cells were selected based on the presence of GFP fluorescence to assess the potassium channel inhibiting activities of the peptides using whole cell patch clamp technique.

### Electrophysiological recordings

An EPC-10 patch-clamp amplifier which was controlled by PatchMaster software (HEKA Elektronik, Lambrecht, Germany) was applied to measure and record the potassium currents for whole-cell. The MPS-2 multichannel microperfusion system (INBIO Inc., Wuhan, China) was applied to exchange the external bath solution with different concentration of peptides. According to the protocol as previously described, the internal patch pipette solution and bath solution were prepared^12^. The peptides were dissolved in Bath solution with 0.01% BSA (Gibco, Pittsburgh, PA, USA) to different concentrations. The potassium currents of hKv1.1, hKv1.2, hKv1.3, hKv1.6 and hKv3.2 channel were generated following the respective protocols as previously described^18^. The results of the electrophysiological experiment are presented as the mean ± SE, and over three cells for each peptide in any concentration were examined (n ± 3).

### The structure analysis of peptides

The 3D structures of BmKcug1a-P1 and its analogs are modelled through the SWISS-MODEL server (https://swissmodel.expasy.org/). The structural diagram of peptides was drawn using Pymol software.

### Circular Dichroism (CD) spectroscopy

The overall structural topologies of BmKcug1-P1, its analogs and mutant peptides in water were analyzed using circular dichroism (CD) spectroscopy with a ChirascanTM V100 spectrometer (Applied Photophysics, Surrey, UK). Each peptide was dissolved in ddH2O at a concentration of 0.2 mg/mL. The instrument’s specific parameter was consistent with those used in a previously published article^13^. At room temperature, the absorption spectra were recorded across a range of wavelengths from 180 to 260 nm for all peptides. Each peptide's absorption spectra were inspected three times. The final CD spectra of each peptide were obtained by averaging the three scans after subtracting the spectrum for ddH_2_O.

### Data analysis

For electrophysiological experiments, we used the ClampFit (Molecular Devices, Sunnyvale, CA, USA) and SigmaPlot software (IBM SPSS, Chicago, IL, USA) to analyze the recorded data. The dose–response relationship between channel current and peptide inhibition is fitted by the modified Hill equation: I_peptide_/I_control_ = 1/{1 + [peptide]/IC_50_}, in which Ipeptide represent the potassium current under the inhibition of peptide, I_control_ represent the peak current, IC_50_ is the half-maximum inhibition concentration, and [peptide] represents the concentration of the peptide. The half inhibitory concentration (IC_50_) was the parameter to be fitted.

## Data Availability

The mass spectrometry proteomics data and Proteome Discover output result file (20,170,902-YF-2-Toxin-8-sensitive.raw) have been deposited to the ProteomeXchange Consortium via iProX (https://www.iprox.cn/page/subproject.html?id=IPX0001470001).
